# Travel history among persons infected with SARS-CoV-2 variants of concern in the United States, December 2020—February 2021

**DOI:** 10.1371/journal.pgph.0001252

**Published:** 2023-03-29

**Authors:** Alicia Dunajcik, Kambria Haire, Jennifer D. Thomas, Leah F. Moriarty, Yuri Springer, Julie M. Villanueva, Adam MacNeil, Benjamin Silk, Jeffrey B. Nemhauser, Ramona Byrkit, Melanie Taylor, Krista Queen, Suxiang Tong, Justin Lee, Dhwani Batra, Clinton Paden, Tiffany Henderson, Audrey Kunkes, Mojisola Ojo, Melanie Firestone, Lindsey Martin Webb, Melissa Freeland, Catherine M. Brown, Thelonious Williams, Krisandra Allen, Judy Kauerauf, Erica Wilson, Seema Jain, Eric McDonald, Elana Silver, Sarah Stous, Debra Wadford, Rachel Radcliffe, Chandra Marriott, Jennifer P. Owes, Stephen M. Bart, Lynn E. Sosa, Kelly Oakeson, Natalie Wodniak, Julia Shaffner, Quanta Brown, Ryan Westergaard, Andrea Salinas, Sara Hallyburton, Yasmin Ogale, Tabatha Offutt-Powell, Kimberly Bonner, Sheri Tubach, Clay Van Houten, Victoria Hughes, Valerie Reeb, Chris Galeazzi, Shreya Khuntia, Sasha McGee, Joseph T. Hicks, Dimple Dinesh Patel, Anna Krueger, Scott Hughes, Fabiana Jeanty, Jade C. Wang, Ellen H. Lee, Tracey Assanah-Deane, Megan Tompkins, Kendra Dougherty, Ozair Naqvi, Matthew Donahue, Justin Frederick, Baha Abdalhamid, Ann M. Powers, Mark Anderson

**Affiliations:** 1 Centers for Disease Control and Prevention (CDC), COVID-19 Response Team, Atlanta, Georgia, United States of America; 2 Michigan Department of Health and Human Services, Lansing, Michigan, United States of America; 3 Georgia Department of Health, Atlanta, Georgia, United States of America; 4 New Jersey Department of Health, Trenton, New Jersey, United States of America; 5 Minnesota Department of Health, St. Paul, Minnesota, United States of America; 6 Epidemic Intelligence Service, CDC, Atlanta, Georgia, United States of America; 7 Colorado Department of Public Health and Environment, Denver, Colorado, United States of America; 8 Texas Department of State Health Services, Austin, Texas, United States of America; 9 Massachusetts Department of Public Health, Boston, Massachusetts, United States of America; 10 Maryland Department of Health, Baltimore, Maryland, United States of America; 11 CDC Foundation, Atlanta, Georgia, United States of America; 12 Washington State Department of Health, Shoreline, Washington, United States of America; 13 Illinois Department of Public Health, Springfield, Illinois, United States of America; 14 North Carolina Department of Health and Human Services, Raleigh, North Carolina, United States of America; 15 California Department of Public Health, Richmond, California, United States of America; 16 San Diego County Health and Human Services Agency, San Diego, California, United States of America; 17 South Carolina Department of Health and Environmental Control, Columbia, South Carolina, United States of America; 18 Pennsylvania Department of Health, Pittsburgh, Pennsylvania, United States of America; 19 Alabama Department of Public Health, Montgomery, Alabama, United States of America; 20 Connecticut Department of Public Health, Hartford, Connecticut, United States of America; 21 Utah Department of Health, Salt Lake City, Utah, United States of America; 22 Virginia Department of Health, Richmond, Virginia, United States of America; 23 Tennessee Department of Health, Nashville, Tennessee, United States of America; 24 Ohio Department of Health, Columbus, Ohio, United States of America; 25 Wisconsin Department of Health Services, Madison, Wisconsin, United States of America; 26 Louisiana Department of Health, New Orleans, Louisiana, United States of America; 27 Indiana Department of Health, Indianapolis, Indiana, United States of America; 28 Delaware Division of Public Health, Dover, Delaware, United States of America; 29 Oregon Health Authority, Portland, Oregon, United States of America; 30 Kansas Department of Health and Environment, Topeka, Kansas, United States of America; 31 Wyoming Department of Health, Cheyenne, Wyoming, United States of America; 32 Southern Nevada Health District, Las Vegas, Nevada, United States of America; 33 Iowa Department of Public Health, Des Moines, Iowa, United States of America; 34 District of Columbia Department of Health (DC Health), Washington, DC, United States of America; 35 New Mexico Department of Health, Santa Fe, New Mexico, United States of America; 36 Kentucky Department for Public Health, Frankfort, Kentucky, United States of America; 37 Maine Center for Disease Control and Prevention, Augusta, Maine, United States of America; 38 New York City Department of Health and Mental Hygiene, New York City, New York, United States of America; 39 Alaska Department of Health and Social Services, Anchorage, Alaska, United States of America; 40 Oklahoma State Department of Health, Oklahoma City, Oklahoma, United States of America; 41 Nebraska Department of Health and Human Services, Lincoln, Nebraska, United States of America; 42 Douglas County Health Department, Omaha, Nebraska, United States of America; 43 Nebraska Public Health Lab, Lincoln, Nebraska, United States of America; University of Minnesota Medical School Twin Cities, UNITED STATES

## Abstract

The first three SARS-CoV-2 phylogenetic lineages classified as variants of concern (VOCs) in the United States (U.S.) from December 15, 2020 to February 28, 2021, Alpha (B.1.1.7), Beta (B.1.351), and Gamma (P.1) lineages, were initially detected internationally. This investigation examined available travel history of coronavirus disease 2019 (COVID-19) cases reported in the U.S. in whom laboratory testing showed one of these initial VOCs. Travel history, demographics, and health outcomes for a convenience sample of persons infected with a SARS-CoV-2 VOC from December 15, 2020 through February 28, 2021 were provided by 35 state and city health departments, and proportion reporting travel was calculated. Of 1,761 confirmed VOC cases analyzed, 1,368 had available data on travel history. Of those with data on travel history, 1,168 (85%) reported no travel preceding laboratory confirmation of SARS-CoV-2 and only 105 (8%) reported international travel during the 30 days preceding a positive SARS-CoV-2 test or symptom onset. International travel was reported by 92/1,304 (7%) of persons infected with the Alpha variant, 7/55 (22%) with Beta, and 5/9 (56%) with Gamma. Of the first three SARS-CoV-2 lineages designated as VOCs in the U.S., international travel was common only among the few Gamma cases. Most persons infected with Alpha and Beta variant reported no travel history, therefore, community transmission of these VOCs was likely common in the U.S. by March 2021. These findings underscore the importance of global surveillance using whole genome sequencing to detect and inform mitigation strategies for emerging SARS-CoV-2 VOCs.

## Introduction

SARS-CoV-2 variants of concern (VOCs) are designated by the Centers for Disease Control and Prevention (CDC), in coordination with the United States (U.S.) Government SARS-CoV-2 Interest Group. VOCs are designated and re-classified as transmission dynamics change based on evidence suggesting increased transmission or coronavirus disease 2019 (COVID-19) severity, impact on diagnostics or therapeutics, or mutations that may allow these variants to evade natural and vaccine-induced immunity more than wild type SARS-CoV-2 [1–9]. As of February 28, 2021, three SARS-CoV-2 phylogenetic lineages were classified as VOCs by CDC: the World Health Organization (WHO)-labeled Alpha variant (Pango lineage B.1.1.7, first detected in the United Kingdom in November 2020), the Beta variant (Pango lineage B.1.351, first detected in South Africa in November 2020), and the Gamma variant (Pango lineage P.1, first detected in Japan via travel from Brazil in January 2021) [9]. By December 2020, the Alpha variant was the dominant SARS-CoV-2 lineage in the United Kingdom and accounted for nearly all cases in the U.K. [10]. By December 2020, the Beta variant had also become dominant in many regions of South Africa and had also begun spreading rapidly to neighboring countries [11, 12]. By January 2021, Gamma was the dominant SARS-CoV-2 lineage in the Amazonas state of Brazil, where Gamma was first detected [13]. These VOCs were identified in the U.S. beginning in December 2020 [9]. This investigation analyzed cases of COVID-19 with whole genome sequencing results showing one of the first three VOCs, reported from December 15, 2020 to February 28, 2021, to determine the proportion of cases with domestic or international travel history in the 30 days preceding a positive SARS-CoV-2 test.

## Materials and methods

Beginning in November 2020, CDC’s National SARS-CoV-2 Strain Surveillance (NS3) system began routine specimen collection from state public health laboratories to generate a nationally representative set of SARS-CoV-2 sequences for genomic surveillance [14]. Cases of COVID-19 attributed to a VOC were also identified through contracts for sequencing data from commercial labs, academic centers and universities, and state and local laboratories [15]. Cases of COVID-19 of Alpha, Beta or Gamma lineages were identified through genomic sequencing and phylogenetic analysis [15]. For all identified cases of COVID-19 attributed to Alpha, Beta, and Gamma lineages, states sent national case surveillance identification (ID) numbers to CDC to enumerate persons infected with VOCs [16].

For this investigation, de-identified lists of COVID-19 cases and their travel history were requested from state and local health departments, with the first request including cases reported from December 15, 2020 to January 28, 2021 and a subsequent request including cases reported from December 15, 2020 to February 28, 2021. Demographic data, including age, sex, race, and ethnicity, were provided by the health jurisdiction or obtained from the Data Collection and Integration for Public Health Event Response system (DCIPHER) using case surveillance ID numbers to link to each reported case if not provided. Travel included out-of-state and international travel within 30 days preceding a positive test for SARS-CoV-2 or COVID-19 symptom onset; persons with unspecified travel dates (n = 31) were assumed to have traveled within this 30-day period and were included in analysis. International travel history for persons infected with a VOC was characterized using the WHO region(s) visited, and whether travel location(s) included the U.K., South Africa, or Brazil [17]. Travel locations, grouped as domestic, international, and both, were compared to the reference group of no travel using a linear trend test over time. For the linear trend test over time, each week was counted as a single time interval, and the assumption of equal time steps was made. A p-value of < .05 was used as the threshold for statistical meaningfulness. Data were analyzed using SAS statistical software (version 9.4, SAS Institute). This activity was reviewed by CDC and was conducted consistent with applicable federal law and CDC policy [18]. Participant data were de-identified before being provided to CDC; therefore, informed consent was not obtained for this investigation. Data from this manuscript are anonymous, as jurisdictions who contributed own their respective data [S1 Table].

## Results

Thirty-five jurisdictions responded to either or both data requests, and data for 1,761 cases of COVID-19 with a VOC on laboratory testing were provided for this investigation. Alpha accounted for 96% (n = 1,684) of cases in the investigation. A substantial proportion of persons infected with a VOC were adults aged 18–29 years (27%) and White (48%). Hospitalization occurred for 5% of cases and 1% died (Table 1).

**Table 1:** Demographic and clinical characteristics of U.S. persons infected with SARS-CoV-2 variants of concern, December 15, 2020-February 28, 2021^a^.

Characteristic	No. (%)			
	All Variants (n = 1761)	B.1.1.7 (n = 1684 [96%])	B.1.351 (n = 68 [4%])	P.1 (n = 9 [<1%])
Sex				
Male	842 (48%)	807 (48%)	32 (47%)	3 (33%)
Female	839 (48%)	802 (48%)	33 (49%)	4 (44%)
Unknown	80 (4%)	75 (4%)	3 (4%)	2 (23%)
Age (years)				
<5	18 (1%)	16 (1%)	2 (3%)	0 (0%)
5–17	213 (12%)	205 (12%)	7 (10%)	1 (11%)
18–29	467 (27%)	450 (27%)	16 (24%)	1 (11%)
30–39	292 (17%)	283 (17%)	9 (13%)	0 (0%)
40–49	262 (15%)	249 (15%)	13 (19%)	0 (0%)
50–64	290 (17%)	277 (16%)	10 (15%)	3 (33%)
65–74	95 (5%)	91 (5%)	4 (6%)	0 (0%)
75–84	37 (2%)	31 (2%)	4 (6%)	2 (22%)
85+	12 (<1%)	11 (1%)	1 (1%)	0 (0%)
Unknown	75 (4%)	71 (4%)	2 (3%)	2 (23%)
Race				
Asian	99 (6%)	98 (6%)	1 (1%)	0 (0%)
American Indian / Alaskan Native	5 (<1%)	4 (<1%)	0 (0%)	0 (0%)
Native Hawaiian / Pacific Islander	2 (<1%)	2 (<1%)	0 (0%)	0 (0%)
Black	371 (21%)	341 (20%)	30 (44%)	0 (0%)
White	845 (48%)	823 (49%)	18 (27%)	2 (22%)
Other	187 (11%)	177 (11%)	8 (12%)	1 (11%)
Unknown	252 (14%)	239 (14%)	11 (16%)	6 (67%)
Ethnicity				
Hispanic	211 (12%)	202 (12%)	7 (10%)	2 (22%)
Not Hispanic	1140 (65%)	1094 (65%)	43 (63%)	3 (33%)
Unknown	410 (23%)	388 (23%)	18 (27%)	4 (45%)
Travel History^b^				
Yes, International Only	85 (5%)	73 (4%)	7 (10%)	5 (56%)
Yes, Domestic Only	91 (5%)	86 (5%)	4 (6%)	1 (11%)
Yes, International and Domestic	20 (1%)	19 (1%)	1 (2%)	0 (0%)
Yes, Location Unknown	4 (<1%)	4 (<1%)	0 (0%)	0 (0%)
No Travel Reported	1168 (66%)	1122 (67%)	43 (63%)	3 (33%)
Unknown	393 (23%)	380 (23%)	13 (19%)	0 (0%)
Visited Presumed VOC Country-of-Origin^c^^,^^d^	n = 105	n = 92	n = 8	n = 5
Yes	21 (20%)	13 (14%)	3 (37%)	5 (100%)
No	84 (80%)	79 (86%)	5 (63%)	0 (0%)
World Health Organization Region(s) Visited^c^	n = 105	n = 92	n = 8	n = 5
Africa	28 (27%)	25 (27%)	3 (37%)	0 (0%)
Eastern Mediterranean	21 (20%)	21 (23%)	0 (0%)	0 (0%)
Americas	20 (19%)	15 (16%)	0 (0%)	5 (100%)
Europe	17 (16%)	17 (19%)	0 (0%)	0 (0%)
Western Pacific	2 (2%)	2 (2%)	0 (0%)	0 (0%)
South-East Asia	1 (1%)	1 (1%)	0 (0%)	0 (0%)
Multiple Regions	16 (15%)	11 (12%)	5 (63%)	0 (0%)
Diagnostic Test for SARS-CoV-2 Identification				
Nucleic Acid Amplification Test	1712 (97%)	1636 (97%)	68 (100%)	8 (89%)
Antigen	47 (3%)	46 (3%)	0 (0%)	1 (11%)
Both Nucleic Acid & Antigen	2 (<1%)	2 (<1%)	0 (0%)	0 (0%)
COVID-19 Symptoms Reported				
Yes	1259 (71%)	1196 (71%)	54 (80%)	9 (100%)
No	124 (7%)	119 (7%)	5 (7%)	0 (0%)
Unknown	378 (22%)	369 (22%)	9 (13%)	0 (0%)
Hospitalization				
Yes	93 (5%)	82 (5%)	8 (12%)	3 (33%)
No	1284 (73%)	1226 (73%)	53 (78%)	5 (56%)
Unknown	384 (22%)	376 (22%)	7 (10%)	1 (11%)
Death				
Yes	19 (1%)	13 (<1%)	5 (7%)	1 (11%)
No	1574 (89%)	1513 (90%)	53 (78%)	8 (89%)
Unknown	168 (10%)	158 (9%)	10 (15%)	0 (0%)

^a^COVID-19 cases attributable to a VOC voluntarily reported to CDC. Data are pooled from 35 public health jurisdictions.

^b^Self-reported travel before symptom onset or positive SARS-CoV-2 test. Persons with unknown travel dates and reported travel within 30 days of a positive SARS-CoV-2 test result were included.

^c^Among persons reporting any international travel history

^d^Country of initial VOC detection: B.1.1.7, United Kingdom; B.1.351, South Africa; P.1, Brazil.

Of 1,761 cases, 1,368 (78%) had available travel history. Among the 1,368 persons with travel data, 1,168 (85%) reported no international or domestic travel during the 30 days preceding a positive SARS-CoV-2 test or symptom onset. 105 individuals (8%) with travel data reported international travel (international travel only or both domestic and international travel in the preceding 30 days), 91 (7%) reported domestic travel only, and four (<1%) did not specify if their travel was domestic or international. For persons infected with Alpha, 1,122 (86%) of 1,304 persons with available travel history reported no travel, 92 (7%) reported international travel, 86 (7%) reported domestic travel only, and four (<1%) reported travel without specifying location. For persons infected with Beta, 43 (78%) of 55 persons with available travel history reported no travel, eight (15%) reported international travel, and four (7%) reported domestic travel only. For the nine persons infected with Gamma, three (33%) reported no travel, five (56%) reported international travel and one (11%) reported domestic travel only.

Among persons infected with a VOC who reported international travel (n = 105), 21 (20%) had been in the U.K., South Africa, or Brazil. Among persons infected with Alpha who reported international travel, 13 (14%) reported being in the U.K. Among persons infected with Beta who reported international travel, three (37%) reported being in South Africa. All five persons infected with Gamma who reported international travel reported being in Brazil. Among persons infected with a VOC who reported international travel, the most common regions were Africa (27%), the Eastern Mediterranean (20%), and the Americas (19%).

**Fig 1:**
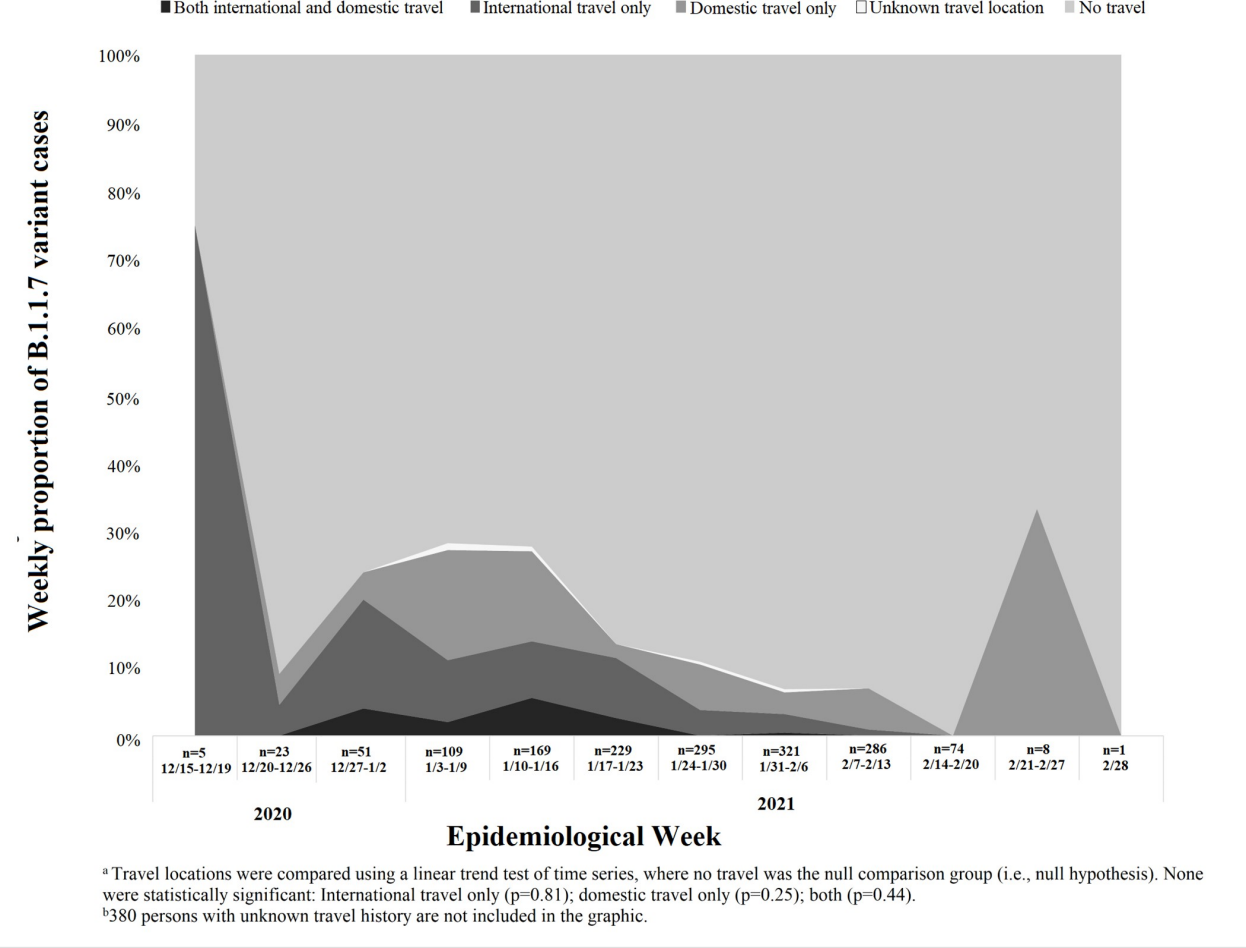
Weekly percent of persons infected with B.1.1.7 SARS-CoV-2 lineage and reported travel by location, December 15, 2020-February 28, 2021^a,b^.

Linear trend tests over time did not show statistically significant differences in trends for international travel (p = 0.81), domestic travel (p = 0.25), or both international and domestic travel (p = 0.44) compared to no travel reported (Fig 1) when using twelve intervals.

## Discussion

Although the first three SARS-CoV-2 lineages designated by CDC as VOCs were initially identified outside of the U.S., few U.S. cases of COVID-19 attributable to these VOCs during November 2020 to March 2021 reported international travel, suggesting widespread transmission in the U.S. during that time period. The detection of persons infected with Alpha in almost all states by February 28, 2021, indicates that transmission of Alpha was widespread, even if travel played a small role in transmission throughout the investigation period [19]. While most persons infected with Alpha and Beta did not report travel, five of the nine persons infected with Gamma reported international travel. The Alpha lineage was the first identified as a VOC in the U.S., and it may have been circulating more widely than subsequently reported Beta and Gamma lineages, which had fewer cases in this investigation.

Prior to February 2021, U.S. genomic surveillance data were limited, which may have led to an inability to detect U.S. introduction of global variants. It is possible that a VOC could have emerged in the U.S. and spread to other countries before being detected [20]. By February 2021, CDC and state and local public health partners had rapidly scaled up the numbers of published sequences from NS3, contracts with commercial laboratories, and other sequencing data sources [19]. Global enhancements to SARS-CoV-2 genomic surveillance would allow for accelerated detection and mitigation of VOCs in the future.

This investigation had several limitations. During the investigation period, genomic surveillance coverage was relatively limited; only a small proportion of SARS-CoV-2 clinical specimens were sequenced. This investigation did not include all cases of COVID-19 known to be due to a a VOC because not all jurisdictions provided data and travel histories were not available for all cases provided. Additionally, findings for all variants may be overpowered by the alpha variant, which had been transmitting the longest and thus had the most cases detected. Because this investigation included travel as a possible VOC exposure for 31 persons with unknown travel dates, some could have been misclassified because their travel was more than 30 days before infection. This 30-day travel window is also longer than the 14-day incubation period for symptomatic SARS-CoV-2, and travel may not have been an exposure for symptomatic patients with onset more than 14 days after travel. However, because only 10 cases occurred more than 14 days after travel, misclassification by dates is assumed to be minimal. A more sensitive definition of travel was selected because this study aimed to rule out travel as a possible explanation of VOC transmission during this study period and including more travel-related cases underscores the limited contributions of travel to VOC transmission.Twenty-three percent of persons in this report had missing travel history; there was also some incompleteness of demographic information due to states not providing demographic data or case IDs provided by states not matching in DCIPHER. Additionally, this study is limited in that household data were not included; and it’s possible that more cases of beta and gamma variants were direct household contacts of someone who traveled. Future research is needed to understand the prevalence of household exposure to travel and how this changes over time as an emerging VOC spreads. Finally, this investigation did not include the WHO-labeled Delta, Pango lineage B.1.617, VOC, first detected in India in October 2020, or the Omicron, Pango lineage B.1.1.529, VOC first detected in South Africa from specimens collected in Botswana in November 2021, as they were designated as VOCs after the investigation period, and were not detected in the U.S. until March 2021 and December 2021 respectively [21–23].

## Conclusions

Of the first three SARS-CoV-2 lineages designated as VOCs in the United States, we found that international travel between December 2020 and February 2021, was common only among a very small number of cases with the Gamma variant (P.1.). The majority of cases infected with either the Alpha or Beta variant reported no travel history, which suggests that widespread transmission of these two VOCs may have been occurring in the United States by February 2021 either due to earlier introduction to the U.S. or emergence within the U.S. These findings underscore the importance of global, systematic whole genome sequencing to detect VOCs before they translocate and to mitigate emerging SARS-CoV-2 VOCs in the U.S. and globally.

VOCs continue to circulate in communities in the U.S. and are sequenced and reported to CDC’s national SARS-CoV-2 genomic surveillance program, which updates the VOC proportions weekly on the CDC website [19]. New variants will likely continue to emerge with potential to spread through travel followed by local transmission, particularly where vaccination rates are low and non-pharmaceutical interventions, including mask adherence, isolation and quarantine, and physical distancing, are not universally adopted.

CDC recommends delaying travel until up to date on COVID-19 vaccines; avoidance of travel, if not up to date on vaccines, may prevent SARS-CoV-2 transmission and translocation of emerging VOCs [24, 25]. CDC recommends mitigating SARS-CoV-2 transmission during travel by following local recommendations and requirements on mask wearing and distancing in a destination, wearing a mask in crowded settings, in indoor public transportation, and when exposed to and/or monitoring for COVID-19 symptoms, and testing and self-isolating if symptomatic or tested positive for SARS-CoV-2 [24]. All travelers are recommended to get tested within three days of departing, and international travelers are also recommended to test 3–5 days after arrival to the United States [25]. Continued scaling up of genomic surveillance may also enable more timely detection of new VOCs globally, potentially limiting their spread [15].

## Supporting information

S1 Table Contact information of jurisdictions providing data. (XLSX)
